# Supplementary material for: Structural and functional characterization of TraI from pKM101 reveals basis for DNA processing
Source: Life Sci Alliance. 2023 Jan 20;6(4):e202201775. doi: 10.26508/lsa.202201775 (PMC9868005; doi:10.26508/lsa.202201775)
Supplement: Supplementary file 1 [file LSA-2022-01775_TableS1.docx]

**Table S1**. List of strains and plasmids used.

| **Strain, plasmid, or oligonucleotide** | **Relevant features or sequences (5’-3’)** | **References** |
| --- | --- | --- |
| **Strains** (*E. coli*): |  |  |
| TOP10 One Shot | Cloning host | Thermo-Fisher |
| BL21 (DE3) | Expression host | Thermo-Fisher |
| **Plasmids:** |  |  |
| P7XC3H | FX cloning expression vector with a C-terminal 10-His-tag | Geertsma & Dutzler, 2011 |
| P7XNH3 | FX cloning expression vector with a N-terminal 10-His-tag | Geertsma & Dutzler, 2011 |
| p7XC3H-TraI | P7XC3H expressing *traI-his_10_* | This study |
| p7XNH3-TraI-H | P7XNH3 expressing *his_10-_traI*_429-910_ | This study |
| p7XC3H-TraI-TE | P7XC3H expressing *traI_1-299_-his_10_* | This study |
| **FX-cloning primers** |  |  |
| Fw-TraI | ATATATGCTCTTCTAGTCTTGATATAACCACGATTACCCGCCAG | Eurofins |
|  |  |  |
| Rv-TraI | TATATAGCTCTTCATGCGATTTCATGGCCCCCTTCTTCATGCTC | Eurofins |
| Rv-TraI-TE | TATATAGCTCTTCATGCTCCCTGCCATTCACGGTTATCAAAATC | Eurofins |
| Fw-TraI-H | ATATATGCTCTTCTAGTCTTAAAAAGACCAGCCACCGCGTCACT | Eurofins |
| Rv-TraI-H | TATATAGCTCTTCATGCGCTCACGCTCGATGCCAAAGATTTTTT | Eurofins |
| ***ss*DNA oligomers** |  |  |
| *oriT57* | TGTGATAGCGTGATTTATCGCGCTGCGTTAGGTGTATAGCAGGTTAAGGGATAAAAA |  |
| F-*oriT*57 | **FITC**-TGTGATAGCGTGATTTATCGCGCTGCGTTAGGTGTATAGCAGGTTAAGGGATAAAAA | Eurofins |
| F*-oriT*35 | **FITC**-TGTGATAGCGTGATTTATCGCGCTGCGTTAGGTGT | Eurofins |
| *F-oriT*20 | **FITC**-TATCGCGCTGCGTTAGGTGT | Eurofins |
| *oriT*11 | GCGTTAGGTGT | Eurofins |
| *oriT*22-F | ATAGCAGGTTAAGGGATAAAAA-**FITC** | Eurofins |
| F-Random57 | **FITC**-TCCGCCATGCAGACGAGACCAGTCGGAGATTACCGAGCATTCTATCAGGTCGGCGAC | Eurofins |
| F-Random35 | **FITC**-CACTAGTGAGCTACTGGAGCCGAGGGGTAACCACG | Eurofins |

Geertsma ER & Dutzler R (2011) A Versatile and Efficient High-Throughput Cloning Tool for Structural Biology. *Biochemistry* 50: 3272–3278
