# Supplementary material for: Structural and functional characterization of TraI from pKM101 reveals basis for DNA processing
Source: Life Sci Alliance. 2023 Jan 20;6(4):e202201775. doi: 10.26508/lsa.202201775 (PMC9868005; doi:10.26508/lsa.202201775)
Supplement: Supplementary file 3 [file LSA-2022-01775_TableS2.docx]

**Table S2**. Data collection and refinement statistics.

| **Data collection summary** | **TraI: DNA-bound** | **TraI: apo-structure** |
| --- | --- | --- |
| Resolution range | 39.67 - 2.1 (2.175 - 2.1) | 45.29 - 1.7 (1.761 - 1.7) |
| Space group | P 21 21 21 | P 21 21 21 |
| Cell dimensions |  |  |
| a, b, c (Å) | 43.444 81.643 90.785 | 40.166 80.628 90.575 |
| α, β, γ (°) | 90 90 90 | 90 90 90 |
| Total reflections | 38982 (3816) | 66280 (6506) |
| Unique reflections | 19496 (1908) | 33141 (3253) |
| Multiplicity | 2.0 (2.0) | 2.0 (2.0) |
| Completeness (%) | 99.85 (99.79) | 99.91 (99.79) |
| Mean I/sigma (I) | 9.16 (1.15) | 12.42 (2.18) |
| R-meas | 0.07283 (0.6719) | 0.03658 (0.4699) |
| CC(1/2)* | 0.996 (0.751) | 0.999 (0.782) |
|  |  |  |
| **Refinement summary** |  |  |
| R-work | 0.2255 (0.3359) | 0.1709 (0.2550) |
| R-free | 0.2695 (0.3514) | 0.1913 (0.2818) |
| Number of non-hydrogen atoms | 2648 | 2594 |
| protein | 2403 | 2376 |
| DNA | 208 |  |
| other ligands | 1 | 42 |
| solvent | 36 | 198 |
| RMS(bonds) | 0.003 | 0.010 |
| RMS(angles) | 0.46 | 1.02 |
| Ramachandran favored (%) | 97.61 | 98.28 |
| Ramachandran allowed (%) | 2.39 | 1.37 |
| Ramachandran outliers (%) | 0.00 | 0.34 |
| Average B-factor | 60.80 | 31.54 |
| protein | 60.50 | 30.73 |
| DNA | 65.97 |  |
| other ligands | 39.89 | 49.90 |
| solvent | 51.46 | 39.37 |

Statistics for the highest-resolution shell are shown in parentheses.
